# How aromatic system size affects the sensitivities of highly energetic molecules?†

**DOI:** 10.1039/d1ra06482g

**Published:** 2021-09-30

**Authors:** Ivana S. Veljković, Jelena I. Radovanović, Dušan Ž. Veljković

**Affiliations:** University of Belgrade – Institute of Chemistry, Technology and Metallurgy, Department of Chemistry Njegoševa 12 11000 Belgrade Republic of Serbia; University of Belgrade – Faculty of Chemistry Studentski trg 12-16 Belgrade 11000 Serbia vdusan@chem.bg.ac.rs

## Abstract

Positive values of electrostatic potentials above the central regions of the molecular surface are strongly related to the high sensitivities of highly energetic molecules. The influence of aromatic system size on the positive values of electrostatic potentials and bond dissociation energies of C–NO_2_ bonds was studied by Density Functional Theory (DFT) calculations on a series of polycyclic nitroaromatic molecules. Calculations performed at PBE/6-311G** level showed that with the increase of the aromatic system size, values of positive electrostatic potential above the central areas of selected energetic molecules decrease from 32.78 kcal mol^−1^ (1,2,4,5-tetranitrobenzene) to 15.28 kcal mol^−1^ (2,3,9,10-tetranitropentacene) leading to the decrease in the sensitivities of these molecules towards detonation. Results of the analysis of electrostatic potential maps were in agreement with the trends in bond dissociation energies calculated for C–NO_2_ bonds of studied nitroaromatic molecules. Bond dissociation energies values indicate that the C–NO_2_ bond in the molecule of 1,2,4,5-tetranitrobenzene (56.72 kcal mol^−1^) is weaker compared to the nitroaromatic molecules with the additional condensed aromatic rings and with a similar arrangement of –NO_2_ groups (59.75 kcal mol^−1^ in the case of 2,3,9,10-tetranitropentacene). The influence of the mutual arrangement of –NO_2_ groups on the sensitivity of nitroaromatic molecules was also analyzed. Results obtained within this study could be of great importance for the development of new classes of highly energetic molecules with lower sensitivity towards detonation.

## Abstract 2

DFT calculations showed that with the increase of the aromatic system size, values of positive electrostatic potential above the central areas of energetic molecules decrease, leading to the decrease in the sensitivities towards detonation.

## Graphical abstract

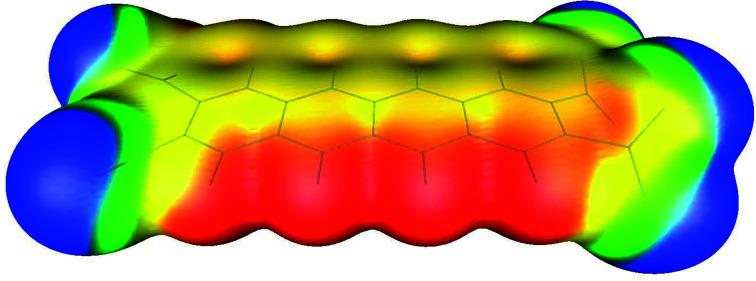

## Introduction

High-energy materials (HEM) are chemical compounds able to store chemical energy and release it upon initiation.^1^ The main disadvantage of many currently available HEM molecules is relatively high sensitivity towards detonation. Search for the new types of energetic molecules with decreased sensitivity towards detonation and satisfactory performance is in the focus of many theoretical and experimental studies.^2–8^ Unfortunately, a balance between high performance and low sensitivity towards detonation is not easy to achieve, since the high efficiency of HEM molecules is usually related to the high impact sensitivity.^1,9,10^ Nitroaromatic molecules are one of the most common classes of highly energetic compounds since they have relatively satisfactory balance between efficiency and sensitivity towards detonation. However, there is still a need for the improvement of the detonation properties of these compounds since the sensitivity of many of them is still very high leading to uncontrolled explosions and industrial accidents. There are three main factors that govern the sensitivity of HEM compounds: (a) maximum detonation heat release, (b) free space per molecule in the crystal lattice and (c) strongly positive electrostatic potential in the middle regions of a molecule.^2,9^ A good indicator of the sensitivity of HEM molecules towards detonation is the value of positive electrostatic potential above the central region of the molecular surface of the energetic molecule.^11–15^ Strong positive potential in the middle regions of nitroaromatic HEM molecules is the consequence of the electron-withdrawing properties of –NO_2_ substituents.^10^ Repulsive interactions between positively charged regions of HEM molecules in crystal lattice increase the resistance to shifting/slipping and make HEM molecules more sensitive. These electron-withdrawing properties are also responsible for the positive potential above C–NO_2_ and N–NO_2_ bonds.^12^ The presence of the positive potential above the C–NO_2_ bonds of nitroaromatic molecules was identified four decades ago in the works of Politzer and co-workers.^12*b*^ These areas of positive electrostatic potential can serve as initial site of nucleophilic attack.^12*c*^

Analysis of molecular electrostatic potential (MEP) was also used to explain the effect of co-crystallization on the detonation properties of many energetic molecules like TNT and CL-20.^8,10^ Analysis of electrostatic potentials of co-crystals containing HEM molecules TNT and CL-20 showed that positive potential in TNT weakens while the positive potential of CL-20 strengthens upon co-crystallization. Results of electrostatic potential analysis of TNT/CL-20 co-crystals were in agreement with experimental results showing that this co-crystal is less sensitive towards detonation than CL-20 compound but more sensitive than TNT molecule.^8,10^

The case of the TNT/CL-20 co-crystals shows that tuning the electrostatic potential values above the centre of the molecular surface of HEM molecules using non-covalent interactions can lead to the design of HEM compounds with lower sensitivities. In our recent work, we showed that hydrogen bonding can be used as a tool in the modification of electrostatic potential values and sensitivities towards detonation of common explosives like 1,3,5-trinitrobenzene, 2,4,6-trinitrophenol, and 2,4,6-trinitrotoluene.^15^ Results of M06/cc-PVDZ calculations showed that in the case when these HEM molecules act as hydrogen atom acceptors positive potential above the central regions of the molecular surface increases up to 10%, while when these molecules act as hydrogen atom donors, positive potential decreases up to 25%.

**Fig. 1:**
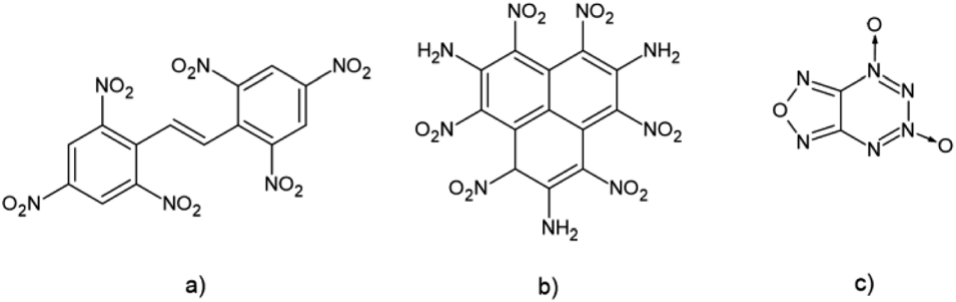
Three-dimensional structure of (a) HNS, (b) HNTAA and (c) FTDO molecules.

It is also known that the introduction of conjugation in molecules of high energetic compounds affects their thermal stability.^16,17^ An obvious example is a molecule of hexanitrostilbene (HNS) which showed to be a very efficient but heat-resistant explosive (Fig. 1). However, in the case of extended conjugation in polycyclic nitroaromatic molecules results are not that unambiguous. These molecules have been studied as potential highly energetic materials with improved detonation properties.^18–20^ An example of a stable polycyclic HEM compound is the HNTAA molecule, a nitroaromatic explosive with three condensed aromatic rings. This HEM compound falls in the group of highly energetic insensitive explosives.^17^

Another example is a group of heterocyclic polyaromatic compounds like tricyclic polyazine *N*-oxides and furazano-1,2,3,4-tetrazine-1,3-dioxide (FTDO).^20^ Unlike the previously mentioned HNTAA explosive, studies showed that these polycyclic HEM compounds are often very sensitive towards detonation. The computational study performed on energetic naphthalene derivatives showed that velocities of detonations of aminonitronaphtalenes are in the range of known secondary explosives and that highly substituted derivatives of naphthalene could be good candidates for the new class of HEM molecules.^21^ An important structural feature that affects the properties of polycyclic nitroaromatic compounds is the size of the condensed aromatic system. In our previous study, we showed that the addition of aromatic rings strengthens C–H/O interactions formed between aromatic hydrocarbons and the water molecule and that the main reason for this is increased polarization which leads to more positive values of electrostatic potentials over hydrogen atoms from C–H fragments.^22^ Since positive values of electrostatic potential in the central regions of HEM molecules are an indicator of their sensitivities towards detonation, changing the aromatic system size could be used as a tool in the design of new polycyclic aromatic HEM molecules.

To elucidate the influence of aromatic system size on the sensitivities toward detonation of polycyclic nitroaromatic compounds, we performed electrostatic potential calculations and bond dissociation energies calculations on a series of tetranitro-derivatives of polycyclic aromatic hydrocarbons with a different number of condensed aromatic rings. The values of the electrostatic potentials in the central regions of these molecules were analysed in the context of their sensitivities towards detonation. We also compared results for the systems with the same number of aromatic rings but the different three-dimensional arrangement of NO_2_ groups, since it was proved that regiochemistry has a significant impact on the properties of high energetic molecules.^23^ Results of the analysis of the electrostatic potentials were combined with the results of bond dissociation energies calculations to establish new rules for the design of polycyclic nitroaromatic HEM molecules with improved detonation properties.

## Methodology

**Fig. 2:**
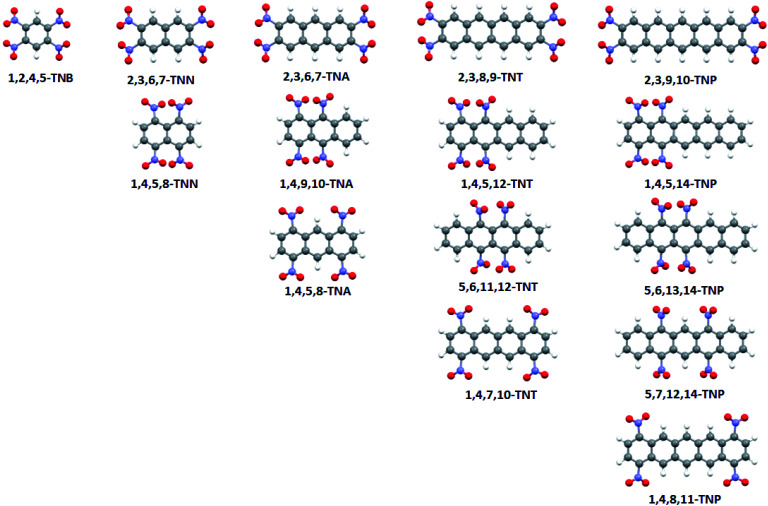
Optimized geometries of 15 studied polycyclic nitroaromatic molecules.

Electrostatic potential maps, wave function files, and Bond Dissociation Energies (BDE) were calculated for optimized geometries of selected nitroaromatic molecules using PBE functional and 6-311G** basis set.^24,25^ Calculations were performed on polycyclic nitroaromatic molecules given in Fig. 2 (IUPAC names of compounds were given in Table 1).

**Table 1:** IUPAC names and abbreviations for the studied nitroaromatic molecules

IUPAC name	Abbreviation
1,2,4,5-Tetranitrobenzene	1,2,4,5-TNB
2,3,6,7-Tetranitronaphthalene	2,3,6,7-TNN
1,4,5,8-Tetranitronaphthalene	1,4,5,8-TNN
2,3,6,7-Tetranitroanthracene	2,3,6,7-TNA
1,4,9,10-Tetranitroanthracene	1,4,9,10-TNA
1,4,5,8-Tetranitroanthracene	1,4,5,8-TNA
2,3,8,9-Tetranitrotetracene	2,3,8,9-TNT
1,4,5,12-Tetranitrotetracene	1,4,5,12-TNT
5,6,11,12-Tetranitrotetracene	5,6,11,12-TNT
1,4,7,10-Tetranitrotetracene	1,4,7,10-TNT
2,3,9,10-Tetranitropentacene	2,3,9,10-TNP
1,4,5,14-Tetranitropentacene	1,4,5,14-TNP
5,6,13,14-Tetranitropentacene	5,6,13,14-TNP
5,7,12,14-Tetranitropentacene	5,7,12,14-TNP
1,4,8,11-Tetranitropentacene	1,4,8,11-TNP

The numbering system in the aromatic molecules was given in Fig. S1.† All calculations were done using Gaussian 09 software package.^26^ Electrostatic potentials were calculated and mapped using the WFA-SAS program.^27^ Bond dissociation energies were calculated according to the procedure previously used for nitroaromatic compounds.^28^ For all structures, geometries were optimized and vibrational spectra calculated. Analysis of the calculated vibrational spectra showed that there were no imaginary frequencies and that optimized geometries are true minima. Three-dimensional structures of molecules were visualized using Mercury software.^29^

## Results and discussion

### Electrostatic potential maps calculations

**Fig. 3:**
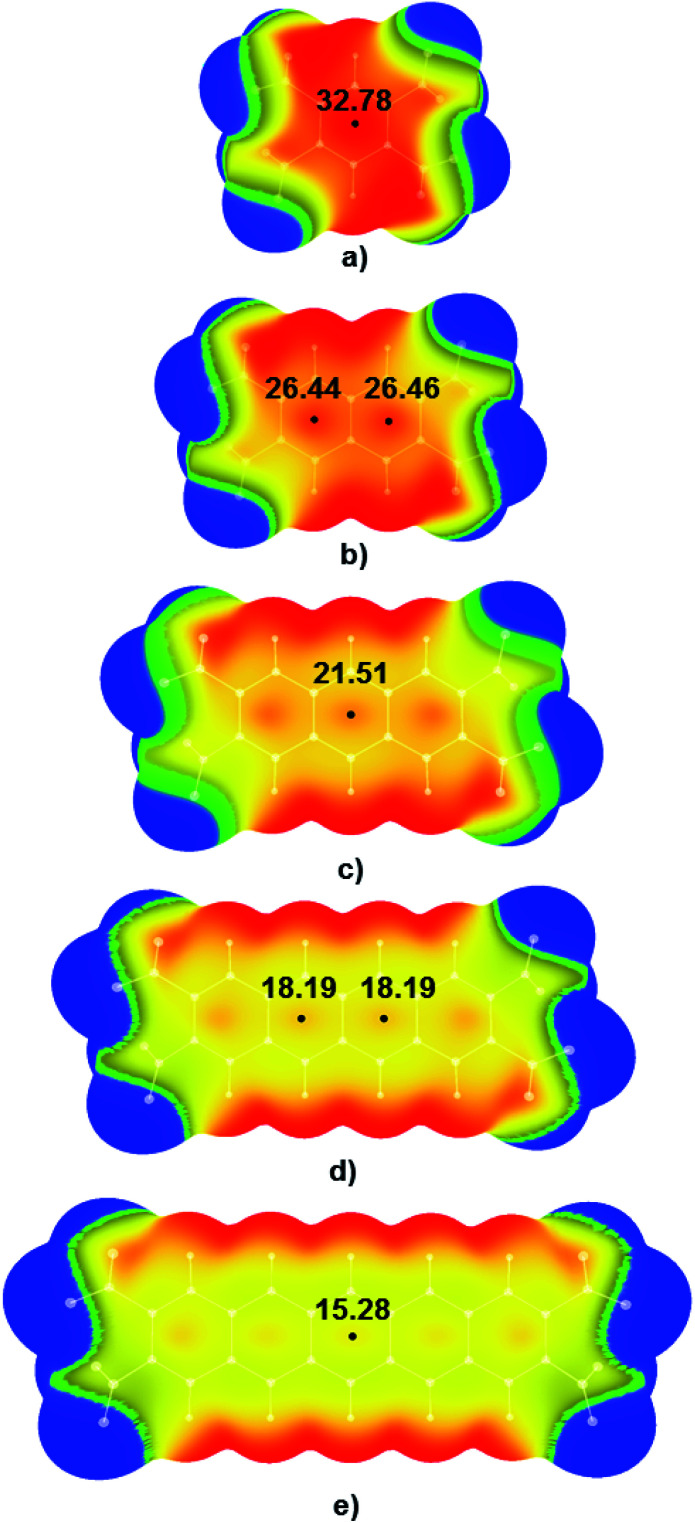
Calculated electrostatic potential maps for (a) 1,2,4,5-TNB, (b) 2,3,6,7-TNN, (c) 2,3,6,7-TNA, (d) 2,3,8,9-TNT and (e) 2,3,9,10-TNP molecule. Values of energies in the critical points are given in kcal mol^−1^. Colour ranges, in kcal mol^−1^, are: red, greater than 25.10; yellow, from 0.00 to 25.10; green, from −2.13 to 0.00; blue, more negative than −2.13. Black dots refer to local maxima on the molecular surfaces.

Electrostatic potential maps were calculated for tetranitro-derivatives of benzene, naphthalene, anthracene, tetracene, and pentacene in which nitro-substituents were located on the ends of the outer rings of linear polycyclic aromatic systems (Fig. 3). Results of DFT calculations show that with the increase of the number of condensed aromatic rings positive values of electrostatic potentials in the central regions of studied nitroaromatic molecules decreases. This decrease is significant; positive potential in the centre of 1,2,4,5-tetranitrobenzene molecule was calculated to be 32.78 kcal mol^−1^, while in the case of 2,3,9,10-tetranitropentacene positive potential decreased approximately to half of the value of the positive potential of 1,2,4,5-tetranitrobenzene (15.28 kcal mol^−1^). This is a decrease in positive electrostatic potential value by 46.61%. It is important to note that another method for controlling electrostatic potential values through hydrogen bonding can modify positive values of electrostatic potentials by up to 25%.^15^ Analysis of electrostatic potential values (Fig. 3) showed that the addition of one condensed aromatic ring lowers positive values of electrostatic potential in the central regions of nitroaromatic molecules by approximately 3-6 kcal mol^−1^. Observed modification of electrostatic potential values in the central regions of polycyclic nitroaromatic molecules could be used as an important tool in the design of new types of explosives with moderate sensitivities towards detonation.

Visual analysis of calculated MEPs showed that in the case of molecules with the most positive values of positive electrostatic potential in the centres of molecules, regions of positive potential could also be identified above the C–NO_2_ bonds (red and yellow areas above the C–NO_2_ bonds in 1,2,4,5-TNB in Fig. 3a). Unlike the 1,2,4,5-TNB, in the case of the 2,3,9,10-TNP molecule with five condensed aromatic rings, there are yellow-green areas of electrostatic potential above the C–NO_2_ bonds.

**Fig. 4:**
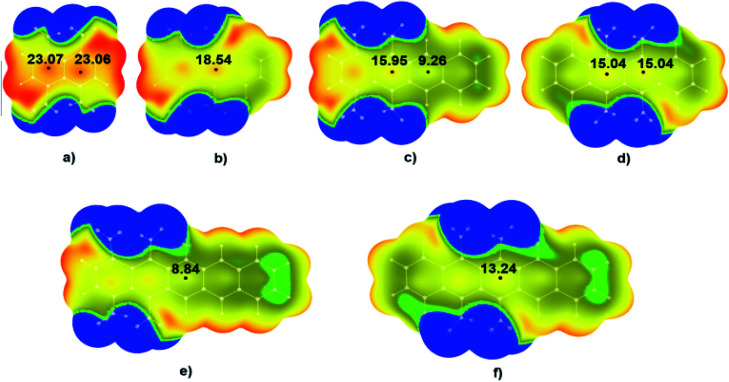
Calculated electrostatic potential maps for polycyclic nitroaromatic molecules with neighbouring –NO_2_ groups: (a) 1,4,5,8-TNN, (b) 1,4,9,10-TNA, (c) 1,4,5,12-TNT, (d) 5,6,11,12-TNT, (e) 1,4,5,14-TNP and (f) 5,6,13,14-TNP. Values of energies in the critical points are given in kcal mol^−1^. Colour ranges, in kcal mol^−1^, are: red, greater than 25.10; yellow, from 0.00 to 25.10; green, from −2.13 to 0.00; blue, more negative than −2.13. Black dots refer to local maxima on the molecular surfaces.

**Fig. 5:**
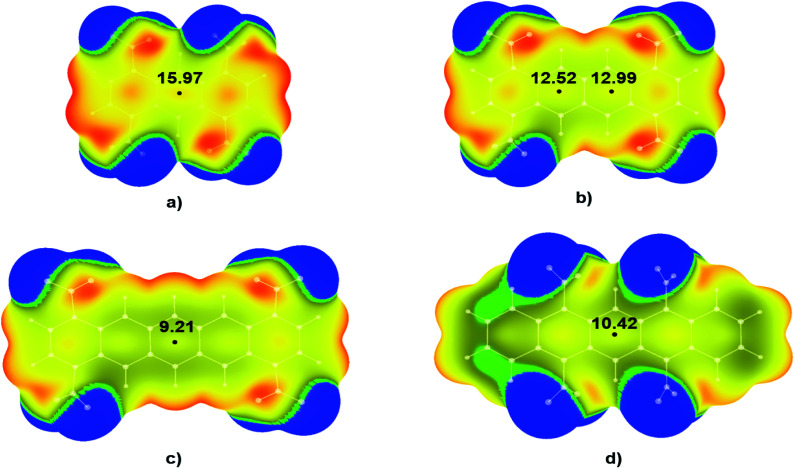
Calculated electrostatic potential maps for polycyclic nitroaromatic molecules with non-neighbouring –NO_2_ groups: (a) 1,4,5,8-TNA, (b) 1,4,7,10-TNT, (c) 1,4,8,11-TNP and (d) 5,7,12,14-TNP. Values of energies in critical points are given in kcal mol^−1^. Colour ranges, in kcal mol^−1^, are: red, greater than 25.10; yellow, from 0.00 to 25.10; green, from −2.13 to 0.00; blue, more negative than −2.13. Black dots refer to local maxima on the molecular surfaces.

To examine the influence of the mutual arrangement of nitro groups on the sensitivities of polycyclic nitroaromatic molecules, MEP were calculated for the tetranitro-derivatives of studied polycyclic molecules in which nitro groups are located on the neighbouring C atoms (Fig. 4) and compared to the MEP of derivatives in which nitro groups were not located on the neighbouring C atoms (Fig. 5).

Results of the DFT calculations showed that also in these molecules values of positive electrostatic potential above the middle regions of studied molecules decreases with the increase of the number of condensed aromatic rings. In the case of molecule 1,4,5,8-TNN (Fig. 4a) values of the positive potential in the two critical points (CPs) in the centre of the molecule were 23.07 kcal mol^−1^ and 23.06 kcal mol^−1^. With the increase of the number of condensed aromatic rings, positive values of electrostatic potentials decrease to 18.54 kcal mol^−1^ in the case of 1,4,9,10-TNA (Fig. 4b), 15.95 and 9.26 kcal mol^−1^ in the case of 1,4,5,12-TNT (Fig. 4c), 15.04 kcal mol^−1^ in the case of 5,6,11,12-TNT (Fig. 4d), 8.84 kcal mol^−1^ in the case of 1,4,5,14-TNP (Fig. 4e) and 13.24 kcal mol^−1^ in the case of 5,6,13,14-TNP (Fig. 4f). It should be pointed out that there is a relatively large difference in the positive potential values of two critical points in the central region of molecule 1,4,5,12-TNT (Fig. 4c). Potential in the critical point in the proximity of –NO_2_ substituents (Fig. 4c, left) was significantly more positive (15.95 kcal mol^−1^) compared to the potential in the other critical point (9.26 kcal mol^−1^) due to the electron-withdrawing effects of –NO_2_ substituents.

Results of the analysis of the electrostatic potentials also showed significant differences between energies in the CPs of two nitroaromatic derivatives of pentacene (Fig. 4e and f). As in the previous case, this is also the consequence of the proximity of the NO_2_ substituents in the molecules 1,4,5,14-TNP and 5,6,13,14-TNP (Fig. 4f).

Visual analysis of the MEPs given in Fig. 4 shows that in the case of the molecules with strong positive potentials in the central regions like 1,4,5,8-TNN (Fig. 4a) there are also areas of positive potential above the C–NO_2_ bonds. On the other hand, in the case of molecules with relatively weak positive potential in the central regions (1,4,5,14-TNP and 5,6,13,14-TNP), there are areas of negative potential above the C–NO_2_ bonds (blue and green areas above C–NO_2_ bonds in Fig. 4e and f).

A similar trend was observed for the tetranitro-derivatives of studied molecules in which nitro groups were not located on the neighbouring C atoms. For the molecule 1,4,5,8-TNA (Fig. 5a) calculated value of electrostatic potential in the central region of the molecular surface was 15.97 kcal mol^−1^, for the molecule 1,4,7,10-TNT (Fig. 5b) 12.52 kcal mol^−1^ and 12.99 kcal mol^−1^, while for the molecules with the largest numbers of condensed aromatic rings values of electrostatic potentials were 9.21 kcal mol^−1^ (1,4,8,11-TNP – Fig. 5c) and 10.42 kcal mol^−1^ (5,7,12,14-TNP – Fig. 5d).

It is important to point out significant differences in the MEPs of isomers with neighbouring and non-neighbouring –NO_2_ groups: isomers with neighbouring –NO_2_ groups have more positive values of electrostatic potentials than isomers with non-neighbouring –NO_2_ groups. For example, values of electrostatic potential in the CP of 1,4,9,10-TNA (Fig. 4b) is 18.54 kcal mol^−1^, which is more positive in comparison to 1,4,5,8-TNA (Fig. 5a) with the electrostatic potential value of 15.97 kcal mol^−1^. Similarly, in the series 5,6,13,14-TNP > 5,7,12,14-TNP > 1,4,8,11-TNP electrostatic potential values in CPs decrease (13.24 kcal mol^−1^ > 10.42 kcal mol^−1^ > 9.21 kcal mol^−1^, respectively). This is consistent with the results of the recent analysis of trigger bonds in nitroaromatic compounds that showed that steric repulsion between neighbouring substituents induce changes in geometries of nitroaromatic compounds leading to the activation of C–NO_2_ trigger bonds.^30^ This structural feature can also be used as a tool in the development of HEM molecules with reduced sensitivities.

The two exceptions were observed in the cases of 1,4,5,12-TNT (Fig. 4c) and 1,4,5,14-TNP (Fig. 4e). In both cases, this anomaly was the consequence of the position of –NO_2_ groups. In 1,4,5,12-TNT molecule, –NO_2_ groups are located on the C atoms further from the centre of the molecule and their electron-withdrawing effect in the central region of the molecule was limited.

In this case, there are significant differences in the values of positive potential between two central CPs so comparison with the MEPs of other isomers was not performed. Differences in the values of positive potential between these two central CPs were the consequence of the distance between –NO_2_ substituents and analysed CPs.

Our results are in agreement with the experimental measurements of the sensitivities of the nitro-substituted benzene and naphthalene derivatives. Experimental impact sensitivity (*h*_50_) value for 1,4,5,8-tetranitronaphthalene was measured to be 100,^31^ while for 1,2,3,5-tetranitrobenzene *h*_50_ value was measured to be 28.^30^ This result indicates that 1,4,5,8-tetranitronaphthalene is less sensitive toward detonation than 1,2,3,5-tetranitrobenzene, which is in agreement with the trends in calculated electrostatic potential values obtained in this study. In the case of trinitro-derivatives of naphthalene and benzene, impact sensitivity of trinitronaphthalene was determined to be 19 N m, while in the case of the 1,3,5-trinitrobenzene is 7.4 N m, indicating that 1,3,5-trinitrobenzene is significantly more sensitive towards impact in comparison to trinitronaphthalene.^32^

In addition to the analysis of the impact sensitivity measurements, we also analyzed electric spark sensitivity (*E*_ES_) measurements for 1,3,5-trinitrobenzene and 1,4,5-trinitronaphthalene molecules. Electric spark sensitivity for 1,3,5-trinitrobenzene was measured to be *E*_ES_ = 6.31 J, while for 1,4,5-trinitronaphthalene electric spark sensitivity was *E*_ES_ = 10.97 J.^33^ These results show that more energy is needed to initiate detonation of nitro-derivatives of naphthalene than nitro-derivatives of benzene with the same number of NO_2_ groups and are in agreement with our results of the analysis of electrostatic potentials.

To compare the influence of the additional aromatic rings with the influence of the additional aliphatic rings on the values of electrostatic potential in the central regions of molecular surface, we calculated electrostatic potential maps for tetranitro-derivatives of polycyclic systems with two, three, four, and five rings in which aromatic rings were located in the centre of the molecule, and aliphatic rings on the sides of the molecules (Fig. S2†). We compared the calculated electrostatic potentials for these systems with the calculated electrostatic potentials of tetranitro derivatives of naphthalene, anthracene, tetracene, and pentacene (Fig. 3).

Analysis of electrostatic potentials shows that in the case of systems containing aliphatic rings, values of electrostatic potential rapidly decrease with the addition of the aliphatic ring (Fig. S3†). Electrostatic potential in the centre of the molecule decreases from 32.78 kcal mol^−1^ in benzene to 15.28 kcal mol^−1^ in the centres of aromatic rings of naphthalene, which is a decrease of Δ*E* = 17.50 kcal mol^−1^.

However, in the case of the system consisting of one aromatic ring in the centre and four aliphatic rings (two on each side of the molecule, Fig. S2d†), electrostatic potential in the centre of the aromatic ring decreases to −5.23 kcal mol^−1^. In this case, the calculated decrease is Δ*E* = 38.01 kcal mol^−1^ and it leads to the negative value of electrostatic potential in the central area of the molecule which indicates that the molecule is insensitive towards detonation. Similar significant decreases were identified in the case of all the other studied systems containing aliphatic rings (Fig. S3†).

### Bond dissociation energy calculations

To additionally examine the influence of the aromatic system size on the sensitivity towards detonation of studied polycyclic nitroaromatic molecules, bond dissociation energies (BDE) were calculated and analysed for the weakest C–NO_2_ bonds (Table 2). Analysis of the BDE (with the zero-potential energy correction) shows that the decrease in the positive electrostatic potential values in the centres of studied HEM molecules is related to the increase in the bond dissociation energies of the weakest bonds in the same molecules.

**Table 2:** Calculated bond dissociation energies with and without zero-point energy correction (ZPE) of selected polycyclic nitroaromatic molecules (Fig. 1)

Compound	Energy of molecule (Hartree)		Energy of fragment (Hartree)		NO_2_ energy (Hartree)		BDE (kcal mol^−1^)	
	Non-corrected	ZPE	Non-corrected	ZPE	Non-corrected	ZPE	Non-corrected	ZPE
1,2,4,5-TNB	−1049.34018	−1049.35642	−844.31353	−844.32700	−204.93515	−204.93903	57.42	**56.72**
2,3,6,7-TNN	−1202.78358	−1202.80247	−997.75489	−997.77108	−204.93515	−204.93903	58.70	**57.96**
1,4,5,8-TNN	−1202.77199	−1202.79058	−997.75070	−997.76668	−204.93515	−204.93903	54.06	53.25
2,3,6,7-TNA	−1356.21827	−1356.23990	−1151.18816	−1151.20713	−204.93515	−204.93903	59.59	**58.82**
1,4,9,10-TNA	−1356.19305	−1356.21453	−1151.17780	−1151.19668	−204.93515	−204.93903	50.26	49.45
1,4,5,8-TNA	−1356.21951	−1356.24116	−1151.18549	−1151.20437	−204.93515	−204.93903	62.04	61.35
2,3,8,9-TNT	−1509.64926	−1509.67369	−1304.61824	−1304.64002	−204.93515	−204.93903	60.16	**59.38**
1,4,5,12-TNT	−1509.62436	−1509.64864	−1304.60701	−1304.62873	−204.93515	−204.93903	51.58	50.75
5,6,11,12-TNT	−1509.61248	−1509.63703	−1304.59590	−1304.61769	−204.93515	−204.93903	51.10	50.39
1,4,7,10-TNT	−1509.65138	−1509.67582	−1304.61673	−1304.63839	−204.93515	−204.93903	62.44	61.74
2,3,9,10-TNP	−1663.078383	−1663.105632	−1458.046759	−1458.071379	−204.93515	−204.93903	60.54	**59.75**
1,4,5,14-TNP	−1663.050179	−1663.077393	−1458.034781	−1458.059354	−204.93515	−204.93903	50.36	49.58
5,6,13,14-TNP	−1663.040814	−1663.068243	−1458.024936	−1458.049624	−204.93515	−204.93903	50.66	49.94
5,7,12,14-TNP	−1663.060391	−1663.088165	−1458.02901	−1458.053913	−204.93515	−204.93903	60.39	59.75
1,4,8,11-TNP	−1663.080284	−1663.107555	−1458.044842	−1458.069406	−204.93515	−204.93903	62.94	62.20

Results showed that among the molecules whose MEPs were given in Fig. 3, C–NO_2_ bonds were weakest in the 1,2,4,5-TNB molecule with bond dissociation energy of 56.72 kcal mol^−1^ (bold ZPE energies in Table 2).

This is the molecule with the strongest positive electrostatic potential above the middle area of the molecular surface (32.78 kcal mol^−1^). BDE values increase in the order: 1,2,4,5-TNB (56.72 kcal mol^−1^) < 2,3,6,7-TNN (57.96 kcal mol^−1^) < 2,3,6,7-TNA (58.82 kcal mol^−1^) < 2,3,8,9-TNT (59.38 kcal mol^−1^) < 2,3,9,10-TNP (59.75 kcal mol^−1^), while in the same order positive values of electrostatic potential in the central regions of studied molecules decreases (Fig. 3).

These results confirm that upon the addition of the condensed aromatic rings in polycyclic nitroaromatic molecules, the energy of C–NO_2_ bonds increases making the bond-breaking process less probable to occur. This also confirms that aromatic system size could be used as a tool for the modification of the sensitivities towards detonation of polycyclic nitroaromatic energetic molecules.

Results of BDE analysis also show that nitroaromatic HEM molecules containing NO_2_ groups on the neighbouring atoms are less stable compared to the HEM molecules containing NO_2_ groups attached to non-neighbouring C atoms. BDE for the weakest C–NO_2_ bond in 1,4,9,10-TNA (NO_2_ substituents on the neighbouring C atoms) molecule was 49.45 kcal mol^−1^, while for the 1,4,5,8-TNA molecule was 61.35 kcal mol^−1^.

Similar trends were calculated for the other studied nitroaromatic molecules (Table 2). Calculated BDE for the molecule 5,6,11,12-TNT (50.39 kcal mol^−1^) indicates that its C–NO_2_ bond is weaker compared to the C–NO_2_ bond in 1,4,7,10-TNT molecule (61.74 kcal mol^−1^). For the nitro-derivatives of the pentacene, BDE for the weakest C–NO_2_ bonds increase in the series: 5,6,13,14-TNP (49.94 kcal mol^−1^) < 5,7,12,14-TNP (59.75 kcal mol^−1^) < 1,4,8,11-TNP (62.20 kcal mol^−1^). In the same order positive potential in the central regions of these molecules and expected impact sensitivity decreases.

Results obtained by bond dissociation energy analysis are consistent with the results of electrostatic potential map analysis indicating that arrangement of –NO_2_ groups in nitroaromatic explosives could be used for the modification of their sensitivities towards detonation.

## Conclusions

Analysis of positive values of molecular electrostatic potential is known to be very useful tool for the assessment of sensitivities of energetic molecules towards detonation. To analyse the influence of the aromatic system size on the sensitivities toward detonation of polycyclic nitroaromatic compounds, values of the electrostatic potentials above the central portion of molecular surfaces were calculated for the series of polycyclic nitroaromatic molecules and discussed in the context of their sensitivities towards detonation. Results of the analysis of calculated electrostatic potentials (Fig. 2) showed that with the increase of the aromatic system size, values of positive electrostatic potential above the central areas of studied highly energetic molecules decrease from 32.78 kcal mol^−1^ (1,2,4,5-tetranitrobenzene) to 15.28 kcal mol^−1^ (2,3,9,10-tetranitropentacene) in the series of linear tetranitro-derivatives of polycyclic aromatic molecules with the NO_2_ groups attached to the ends of outer rings. This decrease in the electrostatic potential values indicates that sensitivities of these molecules towards detonation decrease in the same order. Results of the analysis of the electrostatic potential maps were in agreement with the trends in bond dissociation energies calculated for C–NO_2_ bonds of the same molecules. Calculated bond dissociation energies values indicate that the C–NO_2_ bond in the molecule of 1,2,4,5-tetranitrobenzene (56.72 kcal mol^−1^) is weaker in comparison to the nitroaromatic molecules with the additional condensed aromatic rings and with a similar arrangement of –NO_2_ groups (59.75 kcal mol^−1^ in the case of 2,3,9,10-tetranitropentacene).

Results of calculations also showed that the mutual arrangement of NO_2_ groups strongly affects the sensitivity of nitroaromatic molecules. In the case of molecules with neighboring C–NO_2_ groups in their structures, values of electrostatic potential above the central regions of the molecular surface are more positive compared to the molecules with significant distances between –NO_2_ substituents. Results of bond dissociation energies calculations are consistent with the results of the analysis of electrostatic potentials. BDE values calculated for the weakest C–NO_2_ bonds indicate that strength of these bonds is enhanced upon the addition of the condensed aromatic rings in nitroaromatic energetic molecules.

Results obtained within this study show that aromatic system size could be used as a tool for the modification of the sensitivity towards detonation of nitroaromatic explosives. In the case of nitroaromatic molecules with three or more condensed aromatic rings, changing the mutual arrangement of –NO_2_ groups could provide additional control over the sensitivities of these molecules. These results could be of great importance for the development of new classes of highly energetic materials with improved detonation performances.

## Conflicts of interest

There are no conflicts to declare.

## Supplementary Material

RA-011-D1RA06482G-s001

## References

[cit1] Liu G., Wei S.-H., Zhang C. (2020). Cryst. Growth Des..

[cit2] Politzer P., Murray J. S. (2015). J. Mol. Model..

[cit3] Kent R. V., Wiscons R. A., Sharon P., Grinstein D., Frimer A. A., Matzger A. J. (2018). Cryst. Growth Des..

[cit4] Rice B. M., Hare J. J. (2002). J. Phys. Chem. A.

[cit5] Politzer P., Murray J. S., Grice M. E., Desalvo M., Miller E. (1997). Mol. Phys..

[cit6] Mathieu D. (2018). J. Chem. Inf. Model..

[cit7] Li H., Shu Y., Gao S., Chen L., Ma Q., Ju X. (2013). J. Mol. Model..

[cit8] Zeman S., Jungov M. (2016). Propellants Explos. Pyrotech..

[cit9] Politzer P., Murray J. S. (2016). Propellants, Explos., Pyrotech..

[cit10] Murray J. S., Lane P., Politzer P. (1998). Mol. Phys..

[cit11] Murray J. S., Concha M. C., Politzer P. (2009). Mol. Phys..

[cit12] Politzer P., Murray J. S. (2014). Adv. Quantum Chem..

[cit13] Politzer P., Murray J. S., Koppes W. M., Concha M. C., Lane P. (2009). Cent. Eur. J. Energ. Mater..

[cit14] Matiehu D. (2017). Ind. Eng. Chem. Res..

[cit15] Kretić D. S., Radovanović J. I., Veljković D. Ž. (2021). Phys. Chem. Chem. Phys..

[cit16] Agrawal J. P. (2005). Propellants, Explos., Pyrotech..

[cit17] Liu J., Liu L., Liu X. (2020). Sci. China: Technol. Sci..

[cit18] Türker L., Variş S. (2009). Polycyclic Aromat. Compd..

[cit19] Muthurajan H., Sivabalan R., Talawar M., Anniyappan M., Venugopalan S. (2006). J. Hazard. Mater..

[cit20] Politzer P., Murray J. S. (2015). J. Mol. Model..

[cit21] Murawski R. J., Ball D. W. (2015). Cent. Eur. J. Energ. Mater..

[cit22] Veljković D. Ž. (2018). J. Mol. Graphics Modell..

[cit23] Barton L. M., Edwards J. T., Johnson E. C., Bukowski E. J., Sausa R. C., Byrd E. F. C., Orlicki J. A., Sabatini J. J., Baran P. S. (2019). J. Am. Chem. Soc..

[cit24] Perdew J. P., Burke K., Ernzerhof M. (1996). Phys. Rev. Lett..

[cit25] Krishnan R., Binkley J. S., Seeger R., Pople J. A. (1996). Phys. Rev. Lett..

[cit26] Frisch M. J. , Trucks G. W., Schlegel H. B., Scuseria G. E., Robb M. A., Cheeseman J. R., Scalmani G., Barone V., Mennucci B., Petersson G. A., Nakatsuji H., Caricato M., Li X., Hratchian H. P., Izmaylov A. F., Bloino J., Zheng G., Sonnenberg J. L., Hada M., Ehara M., Toyota K., Fukuda R., Hasegawa J., Ishida M., Nakajima T., Honda Y., Kitao O., Nakai H., Vreven T., Montgomery Jr J. A., Peralta J. E., Ogliaro F., Bearpark M., Heyd J. J., Brothers E., Kudin K. N., Staroverov V. N., Kobayashi R., Normand J., Raghavachari K., Rendell A., Burant J. C., Iyengar S. S., Tomasi J., Cossi M., Rega N., Millam J. M., Klene M., Knox J. E., Cross J. B., Bakken V., Adamo C., Jaramillo J., Gomperts R., Stratmann R. E., Yazyev O., Austin A. J., Cammi R., Pomelli C., Ochterski J. W., Martin R. L., Morokuma K., Zakrzewski V. G., Voth G. A., Salvador P., Dannenberg J. J., Dapprich S., Daniels A. D., Farkas Ö., Foresman J. B., Ortiz J. V., Cioslowski J. and Fox D. J., Gaussian 09, Revision C.01, Gaussian, Inc., Wallingford, CT, 2009

[cit27] Bulat F. A., Toro-Labbe A., Brinck T., Murray J. S., Politzer P. (2010). J. Mol. Model..

[cit28] Rice B. M., Sahu S., Owens F. J. (2002). Journal of Molecular Structure: THEOCHEM.

[cit29] Macrae C. F., Sovago I., Cottrell S. J., Galek P. T. A., McCabe P., Pidcock E., Platings M., Shields G. P., Stevens J. S., Towler M., Wood P. A. (2020). J. Appl. Crystallogr..

[cit30] Shoaf A. L., Bayse C. A. (2018). J. Comput. Chem..

[cit31] Storm C. B. , Stine J. R. and Kramer J. F., Sensitivity Relationships in Energetic Materials, in Chemistry and Physics of Energetic Materials. NATO ASI Series (Series C: Mathematical and Physical Sciences), ed. S.N. Bulusu. Springer, Dordrecht, 1990, ch. 309

[cit32] Meyer R. , Köhler J. and Homburg A., Explosives, Wiley-VCH Verlag GmbH & Co. KGaA, Weinheim, 6th edn, 2007, pp. 348–351

[cit33] Zhi C., Cheng X. (2010). Propellants, Explos., Pyrotech..

