# Supplementary material for: How aromatic system size affects the sensitivities of highly energetic molecules?
Source: RSC Adv. 2021 Sep 30;11(51):31933–40. doi: 10.1039/d1ra06482g (PMC9041559; doi:10.1039/d1ra06482g)
Supplement: RA-011-D1RA06482G-s001 [file RA-011-D1RA06482G-s001.pdf]

Electronic Supplementary Material (ESI) for RSC Advances.
This journal is © The Royal Society of Chemistry 2021

Supporting Information

How aromatic system size affects impact sensitivities of highly energetic molecules?

Ivana S. Veljković, Jelena I. Radovanović, Dušan Ž. Veljković

1. <u>The numbering system in the aromatic molecules</u>

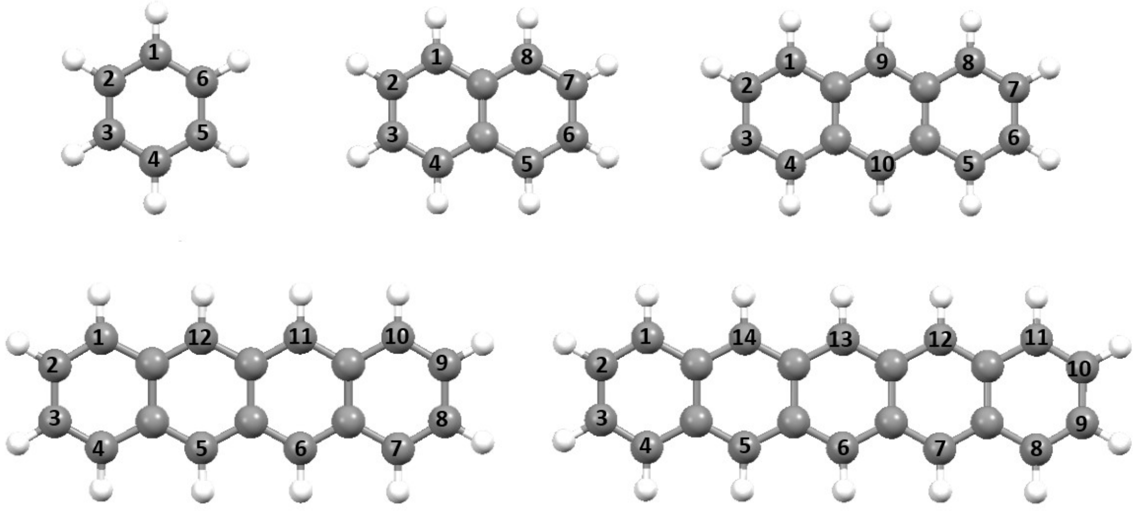

Figure S1. The numbering system in the aromatic molecules studied in this work.

2. <u>Electrostatic Potential Maps for polycyclic nitroaromatic compounds with alifatic rings</u>

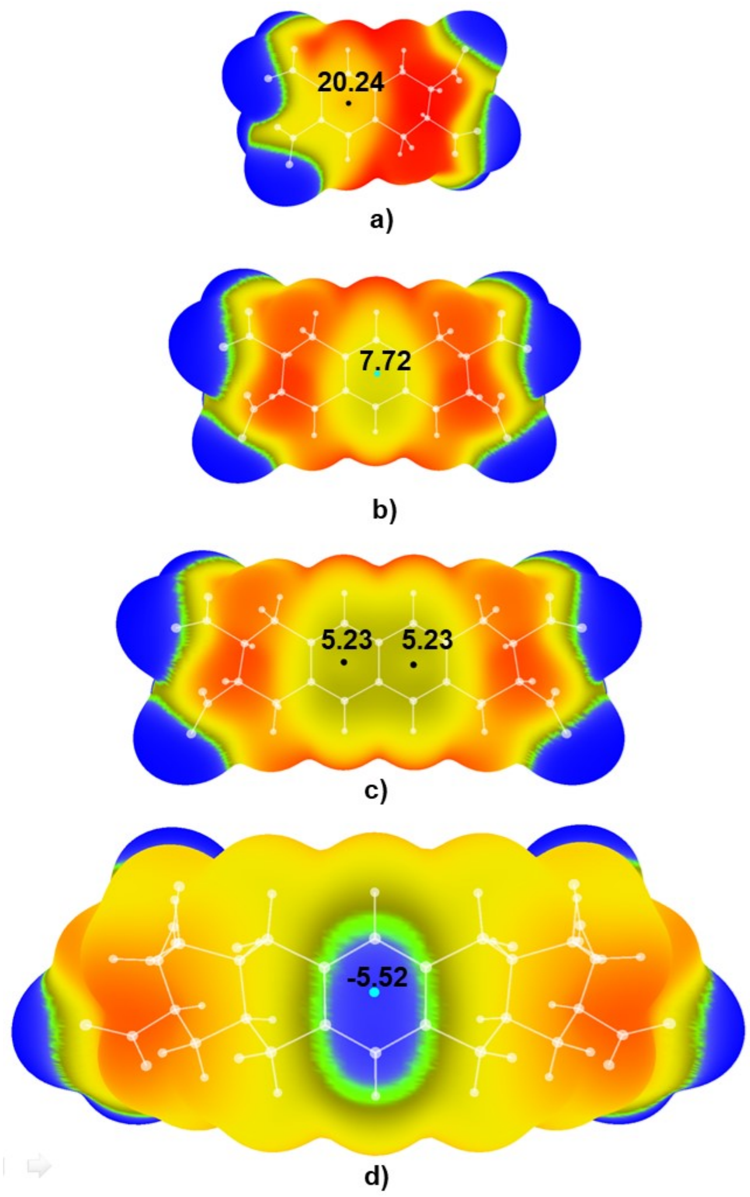

Figure S2. Electrostatic potential maps for the polycyclic nitroaromatic compounds with a) two rings (one aromatic ring (left) and one aliphatic ring (right), b) three rings (one aromatic ring in the center and two aliphatic rings - one on each side), c) four rings (two condensed aromatic rings in the center and two aliphatic rings – one on each side) and d) five rings (one aromatic ring in the center and four aliphatic rings – two on each side). Values of energies in the critical points are given in kcal/mol. Colour ranges, in kcal/mol, are: red, greater than 25.10; yellow, from 0.00 to 25.10; green, from -2.13 to 0.00; blue, more negative than -2.13. Black dots refer to local maxima on the molecular surfaces.

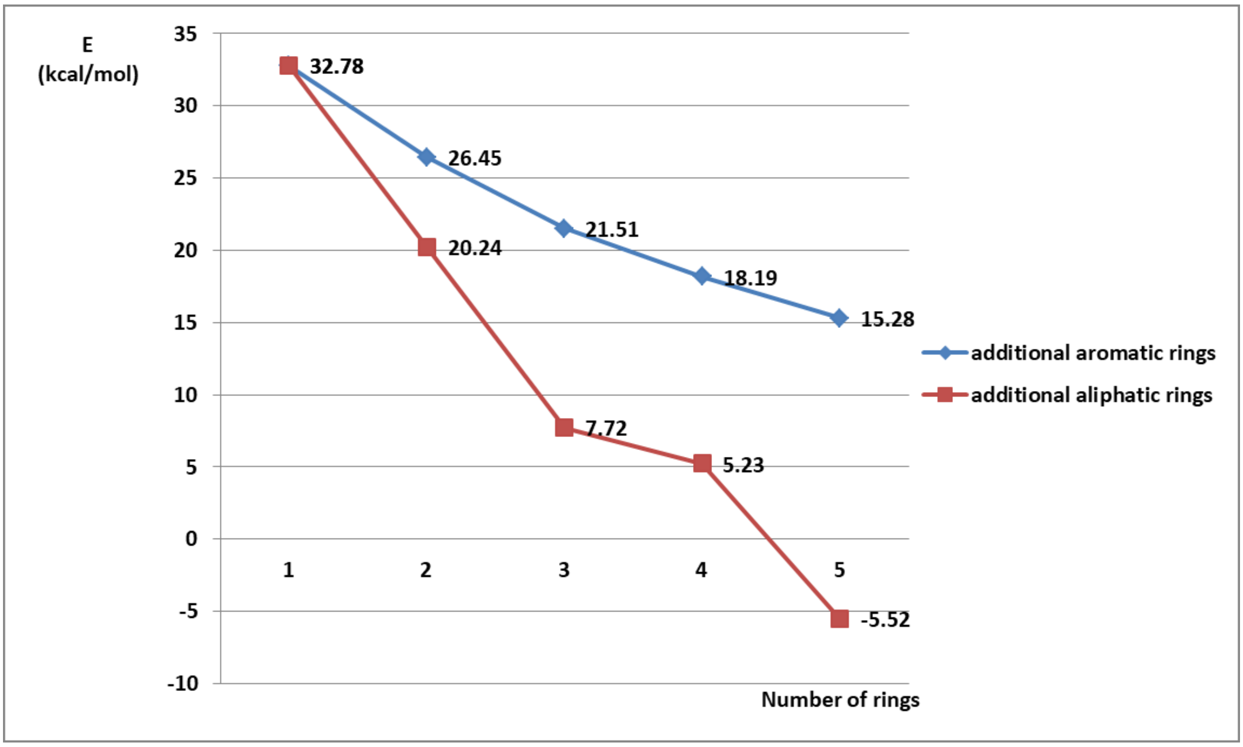

Figure S3. Electrostatic potential maps for the polycyclic nitroaromatic compounds with a) two rings (one aromatic ring and one aliphatic ring), b) three rings (one aromatic ring in the center and two aliphatic rings - one on each side), c) four rings (two condensed aromatic rings in the center and two aliphatic rings – one on each side) and d) five rings (one aromatic ring in the center and four aliphatic rings – two on each side).

3. Cartesian coordinates

Table S1. Cartesian coordinates for 1,2,4,5-tetranitrobenzene

atom	x	y	z
C	0.0000030000	-1.4016970000	0.0000020000
C	-1.2026570000	-0.6999580000	-0.0060310000
C	-1.2026610000	0.7001870000	0.0061150000
C	1.2026600000	-0.6999510000	0.0060350000
C	1.2026530000	0.7001920000	-0.0061190000
C	-0.0000070000	1.4018940000	-0.0000060000
N	-2.4576850000	-1.4986580000	-0.1139160000
O	-3.3021710000	-1.0783320000	-0.8982630000
O	-2.5057540000	-2.5273380000	0.5547630000
N	-2.4577970000	1.4986510000	0.1139410000
O	-2.5048740000	2.5290160000	-0.5522650000
O	-3.3037110000	1.0763660000	0.8957620000
N	2.4577900000	1.4986560000	-0.1139340000
O	3.3037610000	1.0763090000	-0.8956600000
O	2.5048150000	2.5290830000	0.5521790000
N	2.4576920000	-1.4986430000	0.1139100000
O	3.3021910000	-1.0782970000	0.8982320000
O	2.5057510000	-2.5273380000	-0.5547450000
H	-0.0000010000	-2.4924250000	0.0000000000
H	-0.0000040000	2.4926250000	-0.0000100000

Table S2. Cartesian coordinates for 1,4,5,8-tetranitronaphthalene

atom	x	y	z
C	1.3801720000	1.2631390000	-0.1369700000
C	0.7219580000	0.0000130000	0.0000420000
C	-0.7219620000	-0.0000040000	0.0000300000
C	-1.3802030000	1.2631140000	0.1369680000
C	-0.6971180000	2.4582590000	0.0915240000
C	0.6970630000	2.4582730000	-0.0915740000
C	1.3801980000	-1.2631070000	0.1369860000
C	0.6971140000	-2.4582540000	0.0915390000
C	-0.6970700000	-2.4582630000	-0.0915480000
C	-1.3801800000	-1.2631300000	-0.1369430000
N	2.8156080000	1.3660530000	-0.5314110000
O	3.2235170000	0.5107860000	-1.3211920000
O	3.4485970000	2.3303850000	-0.1144300000
N	-2.8156390000	1.3660110000	0.5314040000
O	-3.4486290000	2.3303480000	0.1144310000
O	-3.2235180000	0.5107860000	1.3212460000
N	2.8156460000	-1.3660190000	0.5313860000
O	3.2235550000	-0.5107650000	1.3211770000
O	3.4485970000	-2.3303900000	0.1144490000
N	-2.8156140000	-1.3660380000	-0.5314000000
O	-3.2235020000	-0.5107890000	-1.3212100000
O	-3.4485970000	-2.3304000000	-0.1144740000
H	-1.2557460000	3.3891540000	0.1909210000
H	1.2556650000	3.3891790000	-0.1910160000
H	1.2557400000	-3.3891500000	0.1909270000
H	-1.2556760000	-3.3891660000	-0.1909890000

Table S3. Cartesian coordinates for 2,3,6,7-tetranitronaphthalene

atom	x	y	z
C	1.2397860000	1.4098800000	-0.0080400000
C	-0.0000330000	0.7189650000	-0.0000450000
C	-0.0000200000	-0.7189040000	0.0000380000
C	1.2398090000	-1.4098160000	0.0080480000
C	2.4254550000	-0.7091570000	0.0113610000
C	2.4254420000	0.7092250000	-0.0113830000
C	-1.2398630000	1.4098730000	0.0079160000
C	-2.4255070000	0.7092150000	0.0113420000
C	-2.4254900000	-0.7091890000	-0.0113180000
C	-1.2398450000	-1.4098320000	-0.0079180000
N	-3.6824400000	1.4891350000	0.1829490000
O	-3.7556020000	2.5612260000	-0.4132550000
O	-4.5121220000	1.0146340000	0.9551910000
N	-3.6824430000	-1.4891870000	-0.1829360000
O	-4.5118510000	-1.0150030000	-0.9556520000
O	-3.7557880000	-2.5609910000	0.4136990000
N	3.6823990000	1.4890960000	-0.1829530000
O	3.7556270000	2.5611620000	0.4132510000
O	4.5121300000	1.0145090000	-0.9551190000
N	3.6824690000	-1.4890900000	0.1829470000
O	3.7560090000	-2.5607760000	-0.4138630000
O	4.5118190000	-1.0149290000	0.9557380000
H	1.2718930000	2.5007950000	-0.0243620000
H	1.2718660000	-2.5007290000	0.0243680000
H	-1.2719520000	2.5007920000	0.0241800000
H	-1.2718840000	-2.5007510000	-0.0241790000

Table S4. Cartesian coordinates for 1,4,9,10-tetranitroanthracene

atom	x	y	z
C	-1.3842550000	-1.8668210000	-0.1102230000
C	-0.7269330000	-0.5896740000	0.0025020000
C	-1.3870160000	0.6538870000	0.1248090000
C	-0.7202880000	1.8965690000	0.0946740000
C	-1.3911710000	3.1543900000	0.2333220000
C	-0.6985950000	4.3360110000	0.1276520000
C	0.6984430000	4.3359710000	-0.1290540000
C	1.3910220000	3.1543010000	-0.2341310000
C	0.7201330000	1.8965540000	-0.0949120000
C	1.3869620000	0.6539310000	-0.1244900000
C	0.7269680000	-0.5896760000	-0.0025000000
C	1.3842580000	-1.8668920000	0.1097780000
C	0.7010620000	-3.0567030000	0.0701520000
C	-0.7011000000	-3.0566680000	-0.0710660000
N	-2.8272360000	-2.0029020000	-0.4630380000
O	-3.2507270000	-1.2200360000	-1.3170990000
O	-3.4513440000	-2.9274540000	0.0489440000
N	2.8272090000	-2.0031620000	0.4625560000
O	3.2505480000	-1.2208620000	1.3172160000
O	3.4514130000	-2.9273320000	-0.0499950000
N	-2.8432320000	0.6686430000	0.4428740000
O	-3.5819120000	1.3990990000	-0.2114000000
O	-3.1821280000	-0.0384020000	1.3962680000
N	2.8433960000	0.6690540000	-0.4416010000
O	3.1830220000	-0.0372810000	-1.3952390000
O	3.5814360000	1.3991740000	0.2137520000
H	-1.2316540000	5.2833350000	0.2291830000
H	1.2314720000	5.2832540000	-0.2311080000
H	1.2592590000	-3.9896310000	0.1486250000
H	-1.2592980000	-3.9895550000	-0.1499830000
H	-2.4671470000	3.1724730000	0.3914590000
H	2.4669920000	3.1723750000	-0.3923560000

Table S5. Cartesian coordinates for 1,4,5,8-tetranitroanthracene

atom	x	y	z
C	2.5024240000	1.3924500000	0.0268060000
C	1.2275520000	0.7273320000	0.0192560000
C	0.0001940000	1.4078010000	-0.0000120000
C	-1.2273230000	0.7276440000	-0.0192190000
C	-2.5019520000	1.3932070000	-0.0267670000
C	-3.6933330000	0.7062110000	-0.0009540000
C	-3.6935870000	-0.7049070000	0.0007430000
C	-2.5025080000	-1.3924300000	0.0267310000
C	-1.2275770000	-0.7273770000	0.0192750000
C	-0.0002210000	-1.4078450000	0.0000860000
C	1.2273220000	-0.7277000000	-0.0191650000
C	2.5019960000	-1.3931890000	-0.0266500000
C	3.6933400000	-0.7061040000	-0.0007210000
C	3.6935500000	0.7050160000	0.0009850000
N	2.6237800000	2.8777820000	0.0864990000
O	1.7826370000	3.4980240000	0.7414260000
O	3.5857170000	3.3776260000	-0.4978890000
N	2.6229330000	-2.8785910000	-0.0865740000
O	1.7818360000	-3.4984860000	-0.7418660000
O	3.5845560000	-3.3788790000	0.4979650000
N	-2.6226560000	2.8785530000	-0.0864960000
O	-3.5841700000	3.3788650000	0.4981910000
O	-1.7814890000	3.4983060000	-0.7418580000
N	-2.6240310000	-2.8777460000	0.0865430000
O	-3.5861740000	-3.3775040000	-0.4975890000
O	-1.7828340000	-3.4980600000	0.7413360000
H	0.0003390000	2.4927550000	-0.0000570000
H	-0.0003970000	-2.4927910000	0.0001420000
H	-4.6283630000	1.2658260000	0.0199100000
H	-4.6288100000	-1.2642000000	-0.0201990000
H	4.6283730000	-1.2657030000	0.0201410000
H	4.6287600000	1.2643310000	-0.0198500000

Table S6. Cartesian coordinates for 2,3,6,7-tetranitroanthracene

atom	x	y	z
C	2.4733820000	-1.4107700000	0.0155320000
C	1.2223600000	-0.7244470000	-0.0001500000
C	0.0000350000	-1.4147830000	-0.0000990000
C	-1.2223210000	-0.7244680000	-0.0000310000
C	-2.4732870000	-1.4108520000	-0.0157510000
C	-3.6544310000	-0.7136130000	-0.0161720000
C	-3.6544750000	0.7134530000	0.0160590000
C	-2.4733800000	1.4107650000	0.0155230000
C	-1.2223640000	0.7244380000	-0.0001560000
C	-0.0000300000	1.4147730000	-0.0001010000
C	1.2223180000	0.7244580000	-0.0000200000
C	2.4732940000	1.4108370000	-0.0157150000
C	3.6544360000	0.7136040000	-0.0161500000
C	3.6544800000	-0.7134690000	0.0160760000
N	-4.9075190000	-1.4860660000	-0.2219040000
O	-5.7345680000	-0.9823820000	-0.9802870000
O	-4.9870450000	-2.5825010000	0.3292090000
N	-4.9075350000	1.4859540000	0.2220050000
O	-4.9879580000	2.5814280000	-0.3308240000
O	-5.7334080000	0.9832970000	0.9823220000
N	4.9075450000	-1.4859600000	0.2220100000
O	4.9878750000	-2.5815470000	-0.3306190000
O	5.7335510000	-0.9831600000	0.9820920000
N	4.9075140000	1.4860730000	-0.2218930000
O	5.7344480000	0.9824940000	-0.9804710000
O	4.9870890000	2.5824310000	0.3293620000
H	2.5074010000	-2.5015130000	0.0461050000
H	0.0000260000	-2.5077180000	-0.0001140000
H	-2.5073570000	-2.5015920000	-0.0464410000
H	-2.5073980000	2.5015090000	0.0460860000
H	-0.0000280000	2.5077080000	-0.0001110000
H	2.5073470000	2.5015770000	-0.0463850000

Table S7. Cartesian coordinates for 1,4,5,12-tetranitrotetracene

atom	x	y	z
C	2.6569100000	1.3881170000	0.0185970000
C	1.3732090000	0.7329100000	-0.0150200000
C	0.1342750000	1.3943060000	0.0282420000
C	-1.1201780000	0.7302330000	-0.0362830000
C	-2.3597810000	1.4087040000	-0.0311030000
C	-2.3597480000	-1.4087420000	-0.0307490000
C	-1.1201450000	-0.7302560000	-0.0361880000
C	0.1343150000	-1.3943060000	0.0281540000
C	1.3732370000	-0.7329030000	-0.0150440000
C	2.6569750000	-1.3880360000	0.0186100000
C	3.8303050000	-0.7055000000	0.2193070000
C	3.8302900000	0.7056640000	0.2192730000
C	-4.8312870000	1.4158330000	-0.0356430000
C	-6.0126390000	0.7150240000	-0.0364370000
C	-6.0126180000	-0.7151700000	-0.0361150000
C	-3.5770860000	0.7245860000	-0.0309740000
C	-3.5770620000	-0.7246460000	-0.0307100000
C	-4.8312440000	-1.4159400000	-0.0350490000
N	2.8384340000	-2.8240880000	-0.3351820000
O	3.7021990000	-3.4532640000	0.2695210000
O	2.1543680000	-3.2394680000	-1.2738640000
N	0.1061620000	-2.8524090000	0.3272610000
O	-0.5566000000	-3.5864220000	-0.4018770000
O	0.7293400000	-3.2039740000	1.3323510000
N	2.8382350000	2.8241930000	-0.3352410000
O	3.7019980000	3.4534480000	0.2693690000
O	2.1540790000	3.2395170000	-1.2738920000
N	0.1060800000	2.8523420000	0.3275270000
O	0.7293020000	3.2037950000	1.3326310000
O	-0.5567150000	3.5864470000	-0.4014870000
H	-4.8274600000	2.5084410000	-0.0383350000
H	-4.8273860000	-2.5085480000	-0.0372870000
H	-2.3759160000	2.4972200000	-0.0507470000
H	-2.3759420000	-2.4972600000	-0.0501380000
H	4.7624060000	-1.2672950000	0.2821620000
H	4.7624010000	1.2674440000	0.2820140000
H	-6.9653260000	1.2489330000	-0.0390230000
H	-6.9652900000	-1.2491080000	-0.0384230000

Table S8. Cartesian coordinates for 5,6,11,12-tetranitrotetracene

atom	x	y	z
C	3.7576350000	1.4033240000	-0.1495290000
C	2.4967350000	0.7240390000	-0.0556900000
C	1.2593780000	1.3886240000	-0.0769370000
C	-0.0000030000	0.7310550000	0.0000090000
C	-1.2593940000	1.3886070000	0.0769200000
C	-1.2594100000	-1.3886840000	-0.0768560000
C	0.0000070000	-0.7311440000	0.0000320000
C	1.2594340000	-1.3886630000	0.0769320000
C	2.4967670000	-0.7240180000	0.0555750000
C	3.7577090000	-1.4032320000	0.1493870000
C	4.9373750000	-0.7067610000	0.0843530000
C	4.9373380000	0.7069150000	-0.0845430000
C	-3.7576470000	1.4032840000	0.1494630000
C	-4.9373420000	0.7068610000	0.0844770000
C	-4.9373610000	-0.7068210000	-0.0843720000
C	-2.4967410000	0.7240060000	0.0556770000
C	-2.4967530000	-0.7240550000	-0.0555390000
C	-3.7576880000	-1.4032830000	-0.1493580000
N	1.3206500000	2.8535660000	-0.3353880000
O	1.9619990000	3.5577070000	0.4410640000
O	0.7592640000	3.2323640000	-1.3664550000
N	1.3208320000	-2.8535680000	0.3355710000
O	1.9619010000	-3.5578180000	-0.4410130000
O	0.7597480000	-3.2322460000	1.3668470000
N	-1.3206980000	2.8535590000	0.3353040000
O	-0.7593460000	3.2324140000	1.3663680000
O	-1.9620530000	3.5576490000	-0.4411900000
N	-1.3207750000	-2.8535970000	-0.3354620000
O	-0.7596970000	-3.2322830000	-1.3667380000
O	-1.9618570000	-3.5578390000	0.4411190000
H	3.7772340000	2.4871260000	-0.2451860000
H	3.7773690000	-2.4870300000	0.2450660000
H	5.8851770000	-1.2443260000	0.1517480000
H	5.8851120000	1.2445280000	-0.1519600000
H	-3.7772560000	2.4870900000	0.2450780000
H	-5.8851220000	1.2444680000	0.1518540000
H	-5.8851570000	-1.2443960000	-0.1517730000
H	-3.7773370000	-2.4870830000	-0.2450060000

Table S9. Cartesian coordinates for 1,4,7,10-tetranitrotetracene

atom	x	y	z
C	-3.7347640000	-1.3945850000	0.0001270000
C	-2.4543670000	-0.7346430000	0.0367260000
C	-1.2308440000	-1.4127120000	0.0258930000
C	-0.0013890000	-0.7216590000	0.0128060000
C	1.2279700000	-1.4125180000	-0.0091550000
C	1.2284510000	1.4131010000	0.0107210000
C	-0.0012580000	0.7223550000	0.0164370000
C	-1.2307770000	1.4134300000	0.0293180000
C	-2.4544050000	0.7353540000	0.0363560000
C	-3.7350790000	1.3947660000	-0.0036810000
C	-4.9221260000	0.7062260000	-0.0780590000
C	-4.9220670000	-0.7066960000	-0.0755540000
C	3.7329050000	-1.3953280000	-0.0161920000
C	4.9222880000	-0.7067590000	-0.0182800000
C	4.9221690000	0.7051590000	-0.0634100000
C	2.4519500000	-0.7350310000	-0.0232240000
C	2.4524160000	0.7354630000	-0.0054350000
C	3.7335460000	1.3948040000	-0.0416910000
N	-3.8625770000	-2.8793530000	0.0487990000
O	-3.0213440000	-3.5122470000	0.6935180000
O	-4.8267530000	-3.3738590000	-0.5379930000
N	-3.8637170000	2.8796400000	0.0383770000
O	-4.8272170000	3.3709730000	-0.5523240000
O	-3.0237350000	3.5159950000	0.6812170000
N	3.8628260000	-2.8810830000	-0.0206800000
O	4.8430390000	-3.3537460000	0.5570960000
O	3.0082090000	-3.5371010000	-0.6233460000
N	3.8652260000	2.8804950000	-0.0461260000
O	3.0226170000	3.5407530000	0.5685060000
O	4.8351440000	3.3492010000	-0.6444030000
H	-1.2182510000	-2.5011040000	0.0368050000
H	1.2159070000	-2.5004730000	-0.0371240000
H	1.2174570000	2.5009130000	0.0439250000
H	-1.2183840000	2.5017710000	0.0450290000
H	-5.8559800000	1.2648080000	-0.1372510000
H	-5.8558660000	-1.2656580000	-0.1319940000
H	5.8580580000	-1.2642180000	0.0153530000
H	5.8573460000	1.2619550000	-0.1177150000

Table S10. Cartesian coordinates for 2,3,8,9-tetranitrotetracene

atom	x	y	z
C	3.7055140000	1.4120170000	-0.0185620000
C	2.4504900000	0.7272370000	-0.0003870000
C	1.2346030000	1.4149940000	-0.0030760000
C	-0.0000040000	0.7284940000	-0.0000290000
C	-1.2346060000	1.4149950000	0.0030130000
C	-1.2346030000	-1.4149920000	-0.0030610000
C	-0.0000020000	-0.7284920000	-0.0000240000
C	1.2346060000	-1.4149940000	0.0030200000
C	2.4504920000	-0.7272400000	0.0003420000
C	3.7055260000	-1.4120100000	0.0185220000
C	4.8848070000	-0.7158400000	0.0187250000
C	4.8848120000	0.7158640000	-0.0187420000
C	-3.7055250000	1.4120110000	0.0185230000
C	-4.8848110000	0.7158440000	0.0187300000
C	-4.8848140000	-0.7158600000	-0.0187350000
C	-2.4504970000	0.7272370000	0.0003410000
C	-2.4504950000	-0.7272330000	-0.0003760000
C	-3.7055170000	-1.4120140000	-0.0185470000
N	-6.1347620000	1.4839480000	0.2442580000
O	-6.2174780000	2.5932150000	-0.2816630000
O	-6.9606480000	0.9649070000	0.9947620000
N	6.1347710000	1.4839480000	-0.2442180000
O	6.2173940000	2.5933360000	0.2814780000
O	6.9608310000	0.9647910000	-0.9944520000
N	6.1347620000	-1.4839470000	0.2442500000
O	6.9606090000	-0.9649430000	0.9948190000
O	6.2175030000	-2.5931830000	-0.2817280000
N	-6.1347670000	-1.4839510000	-0.2442260000
O	-6.9607750000	-0.9648370000	-0.9945470000
O	-6.2174220000	-2.5932990000	0.2815450000
H	3.7403460000	2.5025410000	-0.0568970000
H	3.7403510000	-2.5025350000	0.0568350000
H	-3.7403520000	2.5025360000	0.0568400000
H	-3.7403450000	-2.5025380000	-0.0568740000
H	1.2359440000	2.5082780000	-0.0058690000
H	-1.2359420000	2.5082790000	0.0057880000
H	-1.2359430000	-2.5082760000	-0.0058450000
H	1.2359420000	-2.5082780000	0.0058020000

Table S11. Cartesian coordinates for 1,4,5,14-tetranitropentacene

atom	x	y	z
C	5.1337650000	-0.7194850000	0.1241740000
C	5.1337810000	0.7193600000	-0.1240710000
C	6.3935490000	-1.3947390000	0.2401400000
C	3.9178630000	-1.3947240000	0.2406860000
C	6.3935790000	1.3946110000	-0.2399230000
C	3.9178940000	1.3946010000	-0.2406890000
C	7.5743920000	0.7054930000	-0.1210700000
C	7.5743760000	-0.7056260000	0.1213860000
C	2.6813710000	-0.7173330000	0.1243830000
C	2.6813870000	0.7172090000	-0.1244910000
C	1.4551340000	-1.3934670000	0.2247480000
C	1.4551690000	1.3933470000	-0.2249670000
C	0.2226280000	-0.7269370000	0.0964340000
C	0.2226480000	0.7268190000	-0.0967320000
C	-1.0345030000	-1.3920460000	0.1233850000
C	-1.0344110000	1.3920240000	-0.1236940000
C	-2.2667050000	-0.7312490000	-0.0016520000
C	-2.2666710000	0.7313160000	0.0014560000
C	-3.5487370000	-1.3846300000	-0.1353600000
C	-3.5486360000	1.3846880000	0.1356850000
C	-4.7351620000	-0.7012600000	-0.0870670000
C	-4.7350960000	0.7013110000	0.0882110000
N	-1.0274260000	2.8513070000	-0.4220950000
O	-0.2865300000	3.5845510000	0.2281840000
O	-1.7516500000	3.2044750000	-1.3577090000
N	-1.0276760000	-2.8513300000	0.4218910000
O	-1.7519880000	-3.2043610000	1.3574540000
O	-0.2867980000	-3.5846490000	-0.2283200000
N	-3.6855780000	2.8177930000	0.5260160000
O	-2.8907710000	3.2267980000	1.3763580000
O	-4.6218480000	3.4500750000	0.0449150000
N	-3.6858120000	-2.8176070000	-0.5260770000
O	-4.6221260000	-3.4499540000	-0.0450930000
O	-2.8910790000	-3.2264830000	-1.3765340000
H	6.3922520000	-2.4718300000	0.4256260000
H	6.3922990000	2.4717020000	-0.4254060000
H	8.5271240000	1.2317890000	-0.2113680000
H	8.5270960000	-1.2319290000	0.2117670000
H	1.4745680000	-2.4729010000	0.3646600000
H	1.4746170000	2.4727780000	-0.3649230000
H	-5.6686470000	-1.2553050000	-0.1850780000
H	-5.6685280000	1.2553620000	0.1867040000

H	3.9156290000	-2.4728130000	0.4243170000
H	3.9156720000	2.4726920000	-0.4243110000

Table S12. Cartesian coordinates for 5,6,13,14-tetranitropentacene

atom	x	y	z
C	3.2660290000	0.7246230000	-0.0615960000
C	3.2659800000	-0.7248510000	0.0615590000
C	4.5287070000	1.4019000000	-0.1648790000
C	2.0312860000	1.3895050000	-0.0849220000
C	4.5286010000	-1.4022090000	0.1649710000
C	2.0311560000	-1.3896190000	0.0848010000
C	5.7073530000	-0.7066740000	0.0929610000
C	5.7074030000	0.7062730000	-0.0928500000
C	0.7677850000	0.7330980000	0.0004040000
C	0.7677300000	-0.7330750000	-0.0006580000
C	-0.4839780000	1.3912280000	0.0801960000
C	-0.4840830000	-1.3911020000	-0.0804520000
C	-1.7318160000	0.7278810000	0.0558430000
C	-1.7318580000	-0.7276930000	-0.0560900000
C	-2.9744800000	1.4029730000	0.1399440000
C	-2.9744970000	-1.4028040000	-0.1401040000
C	-4.1887520000	0.7219100000	0.0791970000
C	-4.1887690000	-0.7217610000	-0.0792310000
C	-5.4451320000	1.4082230000	0.1539130000
C	-5.4451520000	-1.4080740000	-0.1537790000
C	-6.6253240000	0.7118270000	0.0782360000
C	-6.6253340000	-0.7116780000	-0.0779340000
N	-0.5404200000	-2.8550100000	-0.3380650000
O	-1.1878510000	-3.5615320000	0.4322270000
O	0.0311860000	-3.2350470000	-1.3633290000
N	-0.5401160000	2.8550970000	0.3379190000
O	-1.1872810000	3.5618700000	-0.4323660000
O	0.0313980000	3.2349330000	1.3633200000
N	2.0914980000	-2.8510180000	0.3605100000
O	1.5215090000	-3.2193560000	1.3909620000
O	2.7397550000	-3.5644710000	-0.4021490000
N	2.0918650000	2.8509290000	-0.3603800000
O	2.7402300000	3.5641750000	0.4023720000
O	1.5219040000	3.2195410000	-1.3907610000
H	4.5484890000	2.4845560000	-0.2721650000
H	4.5482720000	-2.4848550000	0.2723550000
H	6.6553380000	-1.2430640000	0.1670730000

H	6.6554230000	1.2426080000	-0.1669270000
H	-7.5782830000	1.2421280000	0.1362010000
H	-7.5783020000	-1.2419790000	-0.1357540000
H	-2.9957810000	2.4888850000	0.2209630000
H	-2.9957340000	-2.4887230000	-0.2211490000
H	-5.4415550000	2.4946220000	0.2706140000
H	-5.4415970000	-2.4944740000	-0.2704780000

Table S13. Cartesian coordinates for 5,7,12,14-tetranitropentacene

atom	x	y	z
C	-3.7195950000	0.7295330000	-0.0206720000
C	-3.7196240000	-0.7295230000	-0.0209800000
C	-4.9804460000	1.4140860000	-0.0487980000
C	-2.4850250000	1.3926360000	-0.0228740000
C	-4.9805220000	-1.4139750000	-0.0495440000
C	-2.4850810000	-1.3926960000	-0.0233520000
C	-6.1584920000	-0.7132140000	-0.0836970000
C	-6.1584610000	0.7134160000	-0.0832890000
C	-1.2278060000	0.7286820000	-0.0276300000
C	-1.2278410000	-0.7287300000	-0.0277430000
C	-0.0000120000	1.4103510000	-0.0000900000
C	0.0000150000	-1.4102570000	-0.0000220000
C	1.2278570000	0.7288180000	0.0275100000
C	1.2277980000	-0.7285750000	0.0276430000
C	2.4851180000	1.3927440000	0.0227940000
C	2.4849650000	-1.3926070000	0.0232950000
C	3.7196670000	0.7295100000	0.0207240000
C	3.7195580000	-0.7295470000	0.0210450000
C	4.9806700000	1.4138030000	0.0490810000
C	4.9803180000	-1.4142550000	0.0496480000
C	6.1585600000	0.7129070000	0.0836170000
C	6.1584150000	-0.7137160000	0.0838860000
N	2.4867670000	-2.8758890000	0.0331040000
O	1.9110220000	-3.4299440000	0.9725940000
O	3.0652920000	-3.4459210000	-0.8938300000
N	2.4870560000	2.8761100000	0.0320290000
O	1.9112000000	3.4306490000	0.9711640000
O	3.0658140000	3.4457650000	-0.8949910000
N	-2.4870180000	-2.8760150000	-0.0331900000
O	-1.9103370000	-3.4301240000	-0.9720930000
O	-3.0664580000	-3.4460220000	0.8932000000
N	-2.4869030000	2.8759960000	-0.0321750000

O	-1.9109280000	3.4303920000	-0.9713100000
O	-3.0655600000	3.4456150000	0.8949370000
H	-4.9949970000	2.5030630000	-0.0326660000
H	-4.9951460000	-2.5029570000	-0.0339820000
H	-7.1075770000	-1.2523070000	-0.1080170000
H	-7.1075240000	1.2525580000	-0.1072740000
H	-0.0000910000	2.4977840000	-0.0001490000
H	0.0000820000	-2.4976960000	-0.0000700000
H	4.9954880000	2.5027660000	0.0330900000
H	4.9947680000	-2.5032430000	0.0340890000
H	7.1076960000	1.2519170000	0.1077420000
H	7.1074230000	-1.2529370000	0.1082010000

Table S14. Cartesian coordinates for 1,4,8,11-tetranitropentacene

atom	x	y	z
C	3.6844330000	-0.7366040000	-0.0316690000
C	3.6844840000	0.7366030000	-0.0331270000
C	4.9665460000	-1.3956220000	0.0101700000
C	2.4633060000	-1.4129650000	-0.0251100000
C	4.9666750000	1.3955790000	0.0084000000
C	2.4633440000	1.4130160000	-0.0287350000
C	6.1529460000	0.7068960000	0.0855050000
C	6.1529010000	-0.7070240000	0.0862150000
C	1.2265550000	-0.7276940000	-0.0171560000
C	1.2265640000	0.7277840000	-0.0195470000
C	0.0012550000	-1.4171080000	-0.0065740000
C	0.0012390000	1.4172200000	-0.0130060000
C	-1.2239910000	-0.7275310000	0.0014300000
C	-1.2240170000	0.7276820000	-0.0025920000
C	-2.4606730000	-1.4126980000	0.0166020000
C	-2.4608110000	1.4128520000	-0.0018700000
C	-3.6822620000	-0.7368190000	0.0232230000
C	-3.6824100000	0.7369610000	0.0075530000
C	-4.9652100000	-1.3959690000	0.0075860000
C	-4.9651900000	1.3959740000	0.0391900000
C	-6.1535700000	-0.7065230000	0.0059990000
C	-6.1533570000	0.7062900000	0.0541940000
N	-5.0984980000	-2.8809750000	0.0056030000
O	-4.2429950000	-3.5434400000	0.6009570000
O	-6.0816910000	-3.3501450000	-0.5708820000
N	-5.0988040000	2.8809530000	0.0449800000
O	-6.0756970000	3.3488610000	0.6332120000

O	-4.2500510000	3.5448070000	-0.5583250000
N	5.0971250000	-2.8797570000	-0.0335900000
O	4.2539930000	-3.5183660000	-0.6711090000
O	6.0647360000	-3.3714980000	0.5507790000
N	5.0975390000	2.8796640000	-0.0363480000
O	4.2545160000	3.5181350000	-0.6741050000
O	6.0652780000	3.3715630000	0.5477430000
H	2.4536140000	-2.5014100000	-0.0369150000
H	2.4537880000	2.5014200000	-0.0435490000
H	7.0869570000	1.2651560000	0.1440270000
H	7.0868840000	-1.2653170000	0.1450170000
H	0.0011570000	-2.5104210000	-0.0054670000
H	0.0011180000	2.5105160000	-0.0187670000
H	-2.4516500000	-2.5006350000	0.0465110000
H	-2.4522800000	2.5007520000	-0.0330290000
H	-7.0895180000	-1.2631580000	-0.0337040000
H	-7.0888380000	1.2627780000	0.1050980000

Table S15. Cartesian coordinates for 2,3,9,10-tetranitropentacene

atom	x	y	z
C	0.7282210000	0.0322720000	-3.6803040000
C	-0.7280260000	-0.0324950000	-3.6802870000
C	1.4119790000	0.0433830000	-4.9372490000
C	1.4149080000	0.0596500000	-2.4676580000
C	-1.4118760000	-0.0432630000	-4.9371820000
C	-1.4146890000	-0.0600080000	-2.4676350000
C	-0.7172930000	-0.0118270000	-6.1159640000
C	0.7172780000	0.0123630000	-6.1159870000
C	0.7299920000	0.0318920000	-1.2279900000
C	-0.7297550000	-0.0322940000	-1.2279860000
C	1.4135410000	0.0623900000	0.0000000000
C	-1.4133070000	-0.0627880000	0.0000000000
C	0.7299920000	0.0318920000	1.2279900000
C	-0.7297550000	-0.0322940000	1.2279860000
C	1.4149080000	0.0596500000	2.4676580000
C	-1.4146890000	-0.0600080000	2.4676350000
C	0.7282210000	0.0322720000	3.6803040000
C	-0.7280260000	-0.0324950000	3.6802870000
C	1.4119790000	0.0433830000	4.9372490000
C	-1.4118760000	-0.0432630000	4.9371820000
C	0.7172780000	0.0123630000	6.1159870000
C	-0.7172930000	-0.0118270000	6.1159640000

N	-1.4932190000	0.1903090000	7.3633680000
O	-2.5865750000	-0.3691350000	7.4462600000
O	-0.9993400000	0.9572990000	8.1903150000
N	1.4930830000	-0.1900040000	7.3634700000
O	2.5866650000	0.3688740000	7.4465020000
O	0.9986710000	-0.9568200000	8.1903220000
N	-1.4932190000	0.1903090000	-7.3633680000
O	-0.9993400000	0.9572990000	-8.1903150000
O	-2.5865750000	-0.3691350000	-7.4462600000
N	1.4930830000	-0.1900040000	-7.3634700000
O	0.9986710000	-0.9568200000	-8.1903220000
O	2.5866650000	0.3688740000	-7.4465020000
H	2.5031620000	0.0491710000	-4.9725650000
H	2.5073390000	0.1051360000	-2.4693890000
H	-2.5030590000	-0.0494450000	-4.9723390000
H	-2.5071260000	-0.1053970000	-2.4693380000
H	2.5060800000	0.1106590000	0.0000000000
H	-2.5058470000	-0.1110330000	0.0000000000
H	2.5073390000	0.1051360000	2.4693890000
H	-2.5071260000	-0.1053970000	2.4693380000
H	2.5031620000	0.0491710000	4.9725650000
H	-2.5030590000	-0.0494450000	4.9723390000
